# Case Report: Telitacicept in severe myasthenia gravis: a case study with multiple autoantibodies

**DOI:** 10.3389/fimmu.2023.1270011

**Published:** 2023-12-06

**Authors:** Qian Guo, Yusen Huang, Fangruyue Wang, Le Fang

**Affiliations:** ^1^ Department of Neurology, China-Japan Union Hospital of Jilin University, Changchun, China; ^2^ The Third Bethune Hospital of Jilin University, Changchun, China

**Keywords:** myasthenia gravis, Titin-Ab, RyR-Ab, AChR-Ab, Telitacicept, case report

## Abstract

Multi-antibody-positive myasthenia gravis (MG) presentations are relatively rare, often found in older patients, and generally predict a poor prognosis. We report a case of a female patient with generalized MG, testing positive for Titin antibodies (Titin-Ab), ryanodine receptor antibodies (RyR-Ab), and acetylcholine receptor antibodies (AChR-Ab), and resistant to acetylcholinesterase inhibitors. Following unsuccessful traditional therapies, she received Telitacicept, leading to significant improvements. This case underscores Telitacicept’s potential efficacy for similar patients and offers insights into the clinical characteristics of multi-antibody MG.

## 1 Introduction

Myasthenia gravis (MG), primarily characterized by muscle weakness and fatigability, a rare autoimmune neuromuscular disorder, in which antibodies target specific proteins involved in the neuromuscular junction function (1). Severe cases may even lead to a life-threatening myasthenic crisis. Although long-term treatment can alleviate symptoms, it often results in side effects and increased disease burden (2–4). A comprehensive meta-analysis showed an MG prevalence of approximately 12.4 cases (95% CI 10.6–14.5) per 100,000 population (5).

In MG patients, the presence of self-reacting antibodies often leads to interference in the communication between nerve and muscle cells (6). These antibodies, produced by a type of immune cell called B-cells, result in muscle weakness and symptoms that typically intensify with repetitive movement. The most common antibody related to MG targets the acetylcholine receptor, known as acetylcholine receptor (AChR). Specifically, thymoma MG patients exhibit high frequencies of AChR antibodies (AChR-Ab, 99.2%) (7).

Furthermore, certain MG patients possess additional autoantibodies, notably those targeting proteins crucial for muscle contraction: Titin and the Ryanodine receptor (RyR) (8, 9). Patients positive for these Titin antibodies (Titin-Ab) and RyR antibodies (RyR-Ab) often display a more severe manifestation of MG. Notably, thymoma MG patients had frequencies of Titin-Ab and RyR-Ab at 50.8% and 46.9% respectively, while late-onset patients showed frequencies of 54.4% and 33.3% (7). The coexistence of Titin-Ab and RyR-Ab may suggest an underlying thymoma. Importantly, these patients tend to exhibit a more severe disease, poorer outcomes, and may require aggressive immunosuppressive treatments. A previous study reported that MG patients with Titin-Ab and RyR-Ab presented with more severe disease symptoms (10).

Telitacicept, like Atacicept, is a transmembrane activator and calcium-modulator and cyclophilin ligand interactor (TACI)-based fusion protein that plays a significant role in regulating B cell activity, crucial in the development of autoimmune diseases. Both Telitacicept and Atacicept aim to mitigate B-cell mediated inflammation; Telitacicept does so by targeting two proteins—B lymphocyte stimulator (BlyS, also known as B cell activating factor, BAFF) and a proliferation-inducing ligand (APRIL)—that are instrumental in B-cell maturation (11). Telitacicept uniquely combines an antibody fragment with a portion of the TACI receptor to inhibit these proteins effectively. While Atacicept was originally designed to treat conditions such as multiple sclerosis and rheumatoid arthritis (12), it was halted in multiple sclerosis due to adverse reactions. Telitacicept, however, with its distinct design, provides a potential treatment to treating a broader spectrum of immune-mediated conditions.

However, to the best of our knowledge, fewer studies have been conducted on this topic. We herein report the clinical presentations, and laboratory characteristics of a patient with MG who was positive for anti-AChR, anti-Titin, and anti-RyR-Abs and well therapeutic response with Telitacicept.

## 2 Case description

A 75-year-old female patient was admitted to our hospital in February 2023. She presented with ptosis that had begun six months prior and had deteriorated one month before admission, along with progressive and fluctuating dysphagia and muscle weakness. In January 2023, she had been definitively diagnosed with MG at an outpatient clinic and had started standard therapy with Pyridostigmine bromide (60 mg, administered thrice daily). Due to progressive deterioration, she increased her dosage of Pyridostigmine bromide to 180 mg, taken 3-4 times per day, resulting in gastrointestinal side effects, including nausea and diarrhea. The patient had a history of coronary heart disease, but there was no family history of neurological disorders.

## 3 Diagnostic assessment

In this report, the patient exhibited characteristic clinical manifestations, including fluctuating ptosis, accompanied by progressive and variable dysphagia and limb weakness. Her quantitative myasthenia gravis(QMG) score was 22. The neostigmine test showed a positive result. The possibility of thymoma or any other tumors was ruled out based on the results of a chest computed tomography (CT) scan and whole-body positron emission tomography (PET-CT). Serology showed positive AChR-Ab (24.10nmol/L, cut off 0.25 nmol/L, ascertained by Radioimmunoassay (RIA)), Titin-Ab (titer 1:300, established through Cell-Based Assay (CBA)), and RyR-Ab (titer 1:30, CBA) and diagnosis of MG was confirmed.

## 4 Therapeutic intervention and follow-up outcomes

Upon hospitalization, the patient was treated with corticosteroids (prednisone 60mg/d, gradually reduced out of hospital) and intravenous immunoglobulin (IVIg 0.4g/kg static for 5 days). The dosage of pyridostigmine bromide was adjusted to 120mg t.i.d and was gradually reduced to 60mg t.i.d. This treatment regime significantly alleviated the patient’s symptoms after two weeks, and her QMG score decreased from 22 to 3.

Given the patient’s advanced age and the potential side effects of long-term corticosteroid and tacrolimus use. Upon stabilization and before hospital discharge, the patient was administered a subcutaneous dose of 160mg of Telitacicept weekly. At the 3-month follow-up, the patient’s AChR-Ab level had reduced to 17.2nmol/L, the RyR-Ab was negative, and the Titin-Ab titer remained stable. Her QMG score had further decreased to 1 at 1-month follow-up and remained at this level throughout the follow-up duration. At the 7-month follow-up, measurements taken on September 5th first indicated an anti-Titin-Ab titer of 1:300, a negative result for the anti-RyR-Ab, and an anti-AChR-Ab concentration of 8.4 nmol/L. Subsequent analysis on September 3rd displayed the patient’s immunoglobulin and complement levels as follows: immunoglobulin A (IgA) was 0.59 g/L, immunoglobulin G (IgG) stood at 8.7 g/L, immunoglobulin M (IgM) was at 0.15 g/L, complement 3 (C3) was 0.72 g/L, and complement 4 (C4) measured 0.2 g/L.

The process of tapering off the steroids was carried out smoothly. The patient has been off medication for over three months and has exhibited no significant adverse reactions.

**Figure 1:**
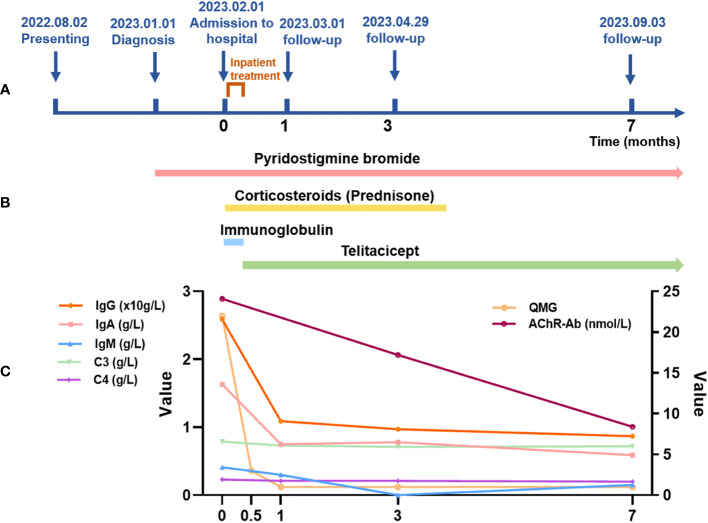
Map of the patient’s course, duration of treatment and changes in immunoglobulins and complement during treatment. **(A)** Map of the patient’s course. The coordinate axis shows the significant milestones from the onset of the patient’s illness to the current follow-up (unit: months), including the onset of the illness, diagnosis, treatment at our hospital, and follow-ups at 1 month, 3 months, and 7 months. The hospitalization for treatment was from February 1, 2023, to February 8, 2023. **(B)** Treatment duration. The arrows indicate the medications used during the patient’s treatment and the duration of use. Pyridostigmine bromide treatment started from the diagnosis of myasthenia gravis (January 1, 2023, to present). Corticosteroid therapy was from hospital admission until the end of May (February 1, 2023, to late May 2023). Immunoglobulin therapy was used during the hospitalization period, lasting 5 days (February 2, 2023, to February 6, 2023). Telitacicept therapy began after the stabilization of the acute phase and continues to present (February 8, 2023, to present). **(C)** Changes in immunoglobulins and complement during the treatment. The line graph shows changes in some data during the patient’s treatment, including changes in immunoglobulin, complement, QMG, and AChR-Ab. Among these, IgG, IgA, IgM, C3, and C4 are on the left vertical axis. The unit for IgG is 10g/L. The units for IgA, IgM, C3, and C4 are g/L. QMG and AChR-Ab are on the right vertical axis. The unit for AChR-Ab is nmol/L. Note: Laboratory Reference Range for Indicators: IgG: 7.51-15.6 g/L; IgA: 0.82-4.53 g/L; IgM: 0.46-3.04 g/L; C3: 0.79-1.52 g/L; C4: 0.16-0.38 g/L; AChR-Ab: positive >0.4 nmol/L, gray area 0.25-0.40 nmol/L, negative<0.25 nmol/L.

Throughout the follow-up period, her clinical condition remained stable, her serum immunoglobulin showed a downward trend (as depicted in **Figure 1**). Both hepatic and renal functions remained within normal ranges, and no infections or other adverse events were observed.

## 5 Discussion

Autoantibodies, such as Titin-Ab and RyR-Ab, are part of the body’s immune response but instead of protecting the body, they attack its cells, tissues, or organs. They play a central role in autoimmune diseases like MG, where they interfere with the transmission of nerve signals to muscles. The complexity of these autoantibodies is derived from their diverse target antigens and the varied clinical manifestations they cause, which may change over time or overlap with other conditions (1). Both Titin-Ab and RyR-Ab belong to the class of striated muscle antibodies, primarily found in the serum of patients with thymoma and late-onset MG (13). Titin-Ab is primarily directed against the MGT-30 molecule expressed by the cDNA sequence located at the junction of the A/I bands on the connectin molecule in Titin, affecting the contraction of the neuromuscular junction and thereby leading to the occurrence of MG (9, 14). The RyR is a transmembrane calcium channel protein playing a pivotal role in calcium ion release and excitation-contraction coupling in skeletal muscle. The presence of RyR-Ab impacts the release of calcium by the RyR, inhibiting the effective contraction of the skeletal muscle and resulting in the development of MG (9).

The detection of Titin-Ab and RyR-Ab is strongly associated with patient age at disease onset and the presence of thymomas, particularly for the latter (7, 15). Some research verifies that Titin and RyR muscle epitopes exhibit a high expression level in cortical thymomas. The concurrent expression of these epitopes with co-stimulatory molecules such as lymphocyte function associated antigen 3 (LFA3, also known as CD58) and BB1 (also known as B7, CD80) on tumor epithelial cells leads to persistent self-sensitization against Titin and RyR within the thymoma, thus fostering T-cell autoimmunity (16). Nevertheless, the patient in the present case did not display any signs of thymoma, thereby excluding thymectomy as a potential treatment strategy. Moreover, the presence of Titin-Ab and RyR-Ab could serve as biomarkers indicative of the disease’s severity, suggesting that patients carrying these antibodies might necessitate more aggressive immunosuppressive therapy (7, 17, 18). This late-onset, generalized MG patient, in our case report, who tested positive for three antibodies but lacked thymoma and showed resistance to acetylcholinesterase inhibitors exhibited swift disease progression. Given this patient’s non-conventional MG condition, choosing an effective immunotherapeutic regimen became critical to our treatment approach.

For remission-phase treatment, expert consensus and data from limited controlled trials support prednisone or prednisolone in combination with azathioprine as the first-line treatment (4, 19). However, azathioprine is characterized by its perceived long onset of action. Furthermore, corticosteroids can easily lead to hypertension and diabetes and exacerbate osteoporosis. Given the advanced age and multiple underlying diseases of the patient in this case, long-term use of corticosteroids may not be tolerable. Tacrolimus is a viable alternative as a second-line immunosuppressive drug (20, 21), but it may cause adverse reactions in multiple systems, including ischemic coronary artery disease, hypertension, and diabetes. The patient, in this case, was of advanced age with atherosclerotic heart disease, and using tacrolimus could increase her risk of cardiovascular disease. Moreover, tacrolimus cannot be arbitrarily reduced or discontinued, offering limited flexibility. Therefore, after thorough communication with the patient and her family, tacrolimus was not chosen as the long-term immunotherapy drug.

B cells play a key role in the pathogenesis of MG, including autoantibody production, cytokine secretion, and antigen presentation to autoreactive T cells. Telitacicept, a novel recombinant fusion protein that targets both BAFF and APRIL, which both are crucial for B-cell maturation and survival (22). By inhibiting these factors, Telitacicept could suppress the production of harmful autoantibodies, a notable characteristic of autoimmune diseases like MG. The well-established role of B-cells and autoantibodies in the pathogenesis of MG substantiates the possible benefits of using B-cell targeting treatments like Telitacicept (23). This approach could aid in the reduction of autoantibody production in MG, thereby alleviating the severity of the disease. Furthermore, existing studies on the use of Telitacicept in other autoimmune diseases have demonstrated promising results. For instance, in the treatment of systemic lupus erythematosus (SLE) (24, 25) and rheumatoid arthritis (RA) (26), Telitacicept has shown considerable effectiveness and safety. This suggests the potential for similar results in MG patients.

In our case report, we noted significant improvement after approximately seven months of treatment with Telitacicept. Furthermore, the symptoms remained stable during the 7-month follow-up period. The patient had been off corticosteroid therapy for more than 3 months, which was a surprising discovery given the high dependence on corticosteroids for patients with MG. The patient’s immunological response to treatment was also indicative of the effectiveness of the therapeutic regime, as evidenced by a decreased concentration of AChR-Ab to 8.4 nmol/L. Additionally, the RyR-Ab test results transitioned to negative, suggesting a successful suppression of this particular autoimmune response. The titer for RyR-Ab remained stable, indicating a controlled state of the disease. In terms of clinical symptoms, the patient’s QMG score – a commonly used index to measure the severity of MG – had reduced to a mere 1, hinting at a significant improvement in her condition. It was also worth noting that during the follow-up period, the patient’s condition remained impressively stable, without any new symptoms or disease flares. Moreover, a downward trend was noticed in the levels of her serum immunoglobulin and complement, two crucial components of the immune system, providing further evidence of the effectiveness of the treatment (as demonstrated in **Figure 1**). Lastly, and perhaps most importantly from a patient’s perspective, no infections or other adverse events were recorded throughout this follow-up period. This underscores the tolerability and safety of the implemented therapeutic strategy (Telitacicept), in addition to its efficacy, and suggests a promising approach to managing similar cases of severe MG.

## 6 Patient perspective

The patient described her journey with MG as challenging. Daily activities were difficult due to muscle weakness, leading to a significant loss of independence. Despite initial treatment, her condition worsened, causing her frustration and emotional distress. Dysphagia, dysarthria, and breathing difficulties amplified her anxiety. However, after admission to our institution and an adjusted treatment plan, she noted a gradual improvement in her symptoms. This relief improved her mood and outlook significantly. The reduced gastrointestinal side effects, thanks to the new treatment, also alleviated some of her illness burdens. The patient expressed satisfaction with the care received at our hospital, the comprehensive explanation of her condition, and the introduction of Telitacicept.

## Data availability statement

The original contributions presented in the study are included in the article/supplementary material. Further inquiries can be directed to the corresponding author.

## Ethics statement

The studies involving humans were approved by Ethics Committee of China-Japan Union Hospital of Jilin University. The studies were conducted in accordance with the local legislation and institutional requirements. The participants provided their written informed consent to participate in this study. Written informed consent was obtained from the individual(s) for the publication of any potentially identifiable images or data included in this article. Informed consent was obtained from the patient for this case report. Additionally, while our hospital's ethics committee exempts single case reports from ethical declarations, the entire study was conducted in strict adherence to ethical standards and the Declaration of Helsinki.

## Author contributions

QG: Visualization, Writing – original draft. YH: Resources, Writing – review & editing. FW: Writing – review & editing. LF: Project administration, Supervision, Writing – review & editing.
